# A Test of the Thermal Melanism Hypothesis in the Wingless Grasshopper *Phaulacridium vittatum*

**DOI:** 10.1673/031.013.5101

**Published:** 2013-06-06

**Authors:** Rebecca M. Harris, Peter McQuillan, Lesley Hughes

**Affiliations:** 1 School of Geography and Environmental Studies, University of Tasmania, Private Bag 78 Hobart, 7001, Australia; 2 Department of Biological Sciences, Macquarie University, North Ryde, NSW, 2109, Australia

**Keywords:** altitude, geographic variation, insects, Orthoptera, reflectance

## Abstract

Altitudinal clines in melanism are generally assumed to reflect the fitness benefits resulting from thermal differences between colour morphs, yet differences in thermal quality are not always discernible. The intra-specific application of the thermal melanism hypothesis was tested in the wingless grasshopper *Phaulacridium vittatum* (Sjöstedt) (Orthoptera: Acrididae) first by measuring the thermal properties of the different colour morphs in the laboratory, and second by testing for differences in average reflectance and spectral characteristics of populations along 14 altitudinal gradients. Correlations between reflectance, body size, and climatic variables were also tested to investigate the underlying causes of clines in melanism. Melanism in *P. vittatum* represents a gradation in colour rather than distinct colour morphs, with reflectance ranging from 2.49 to 5.65%. In unstriped grasshoppers, darker morphs warmed more rapidly than lighter morphs and reached a higher maximum temperature (lower temperature excess). In contrast, significant differences in thermal quality were not found between the colour morphs of striped grasshoppers. In support of the thermal melanism hypothesis, grasshoppers were, on average, darker at higher altitudes, there were differences in the spectral properties of brightness and chroma between high and low altitudes, and temperature variables were significant influences on the average reflectance of female grasshoppers. However, altitudinal gradients do not represent predictable variation in temperature, and the relationship between melanism and altitude was not consistent across all gradients. Grasshoppers generally became darker at altitudes above 800 m a.s.l., but on several gradients reflectance declined with altitude and then increased at the highest altitude.

## Introduction

Melanism, the occurrence of darker pigmentation in individuals within species or between closely related species, is common in many animal groups and well-documented in insects. There are several hypotheses explaining why it has evolved, including mate attraction, crypsis, and resistance to disease, desiccation, and UV radiation (Majerus 1998). The hypothesis that has attracted the most attention is the thermal melanism hypothesis, which states that under conditions of low temperature, dark individuals are at an advantage compared to light individuals because they warm up more quickly at any given level of radiation (Clusella-Trullas et al. 2007; Clusella Trullas et al. 2007). This improved fitness is expressed in higher proportions of dark individuals being found in cold environments, such as at high altitudes.

Increased fitness through thermal melanism has been demonstrated in several insect groups, including butterflies (Roland 1982; Ellers and Boggs 2004; Roland 2006), beetles (Rhamhalinghan 1999), and grasshoppers (Unsicker et al. 2008). Fitness consequences of melanisation include increased activity (Roland 1982; Guppy 1986; de Jong et al. 1996; Ellers and Boggs 2004; Roland 2006), feeding and mating (Brakefield 1984b; Parkash et al. 2008), fecundity and egg maturation rates (Ellers and Boggs 2004), survival (Kingsolver 1995; Parkash et al. 2008), and predator avoidance (Clusella-Trullas et al. 2007). Additional support for the thermal melanism hypothesis is provided by laboratory rearing experiments, in which insects, such as butterflies (Lewis 1985; Marriott and Holloway 1998; Karl et al. 2009) and hoverflies (Marriott and Holloway 1998), reared atlower temperatures are darker than those reared at higher temperatures.

Altitudinal gradients have traditionally been used to test the thermal melanism hypothesis under natural conditions because they represent steep gradients in temperature over relatively short distances, facilitating gene flow and minimising the influence of other covarying factors is (Körner 2007).

While it has been suggested that a positive relationship between altitude and melanism has been well-demonstrated in a range of insects (True 2003; Clusella-Trullas et al. 2007), much of the research has actually focused on very few species, for example *Colias* butterflies (Watt 1968; Roland 1982; Ellers and Boggs 2002), or reports inter-specific comparisons (Watt 1968; Rapoport 1969; Kingsolver 1983; Kingsolver and Watt 1983). Published papers that present tests of altitudinal clines in melanism within species are in fact restricted to one species of Homoptera (Halkka et al. 1980; Berry and Willmer 1986; Boucelham and Raatikainen 1987), three species of Lepidoptera (Ellers and Boggs 2002; Karl et al. 2009; Karl et al. 2010), one species of Diptera (Munjal et al. 1997; Rajpurohit et al. 2008) and one species of Orthoptera (Guerrucci and Voisin 1988). Further, very few of these studies have investigated change along continuous altitudinal gradients. They report general correlations between altitude and colour at unrelated sites (eg. Halkka et al. 1980; Guerrucci and Voisin 1988) or compare the extremes of altitude (Rajpurohit et al. 2008; Karl et al. 2010). Others are studies of extreme habitats, such as alpine, arctic, or subAntarctic habitats (Roland 1978; Mikhailov 2001; Davies et al. 2007). In this paper, differences in melanism in temperate grasshoppers collected from several sitesalong replicated gradients are described. Thus, trends in altitude and temperature, the existence of thresholds, and the influence of site or habitat differences can be identified.

Melanism has been quantified in a number of ways. In species that can be separated into distinct colour morphs, the representation of colour morphs within populations has been compared (Brakefield 1984a; de Jong and Brakefield 1998; Pereboom and Biesmeijer 2003). In other species, the area covered by dark spots (Stewart and Dixon 1989), bands (Honek 1993), or scales (Lewis 1985; Guppy 1986) has been measured. However, these approaches are not possible in species where melanism represents a gradation in colour. A few studies have used spectrometry to quantify average reflectance as a measure of melanism (e.g., Brakefield and Willmer 1985). Here, the colour of grasshoppers along altitudinal gradients is described by quantifying differences in spectral data. A spectrum is a graphical representation of the reflection of light from a sample. As colour changes, the slope of the curve, amplitude, and the number and position of peaks within the spectrum all change (Grill and Rush 2000). These spectral data can also be condensed into three colour components, namely brightness, chroma, and hue. Brightness is a measure of light intensity across all wavelengths, chroma is a measure of the purity of a colour, and hue is colour (e.g., violet, blue, green, red) (Endler 1990; Grill and Rush 2000).

The thermal melanism hypothesis rests on the assumption that there is a relationship between reflectance, body size, and the absorption of radiation. This has been demonstrated under laboratory conditions in many insects, including beetles (Brakefield and Willmer 1985; Stewart and Dixon 1989; de Jong et al. 1996; Rhamhalinghan 1999), bees(Pereboom and Biesmeijer 2003), butterflies (Watt 1968, 1969), and grasshoppers (Forsman 1997). However, it has also been suggested that the difference in body temperature between dark and light individuals would be too small to be of any ecological importance, or that other effects, such as predator avoidance, would swamp any thermoregulatory advantage (Digby 1955; Willmer and Unwin 1981).

The primary objective of this study was to test the intra-specific application of the thermal melanism hypothesis in the wingless grasshopper *Phaulacridium vittatum* (Sjöstedt) (Orthoptera: Acrididae). The hypothesis that there is no difference in the thermal properties of the different colour morphs was tested using laboratory experiments. Then, the hypothesis that the average reflectance and spectral characteristics of populations do not differ along altitudinal gradients was tested. Furthermore, the underlying causes of clines in reflectance and body size was investigated by considering the proportion of winged and striped grasshoppers, and correlations between reflectance, body size, and climatic variables (temperature and rainfall).

*P. vittatum* was used because it is a common, widely-distributed species that has variable body size and is polymorphic for colour pattern. If the thermal melanism hypothesis is supported, the following would be expected: darker individuals warm up and cool down more quickly and reach higher equilibrium temperatures than lighter ones; a greater proportion of dark individuals (i.e. lower reflectance) found at high altitude sites compared with low altitude populations; a negative correlation between melanism and body size; and a negative correlation between melanism and temperature (i.e., positive correlation between reflectance and temperature).

## Methods and Materials

*P. vittatum* is a common species of Acrididae, widely distributed in open habitats in the cool, temperate areas of eastern and southern Australia (-23° 36′ to -43° 0′ S latitude). It is restricted to higher elevations in the north, but its altitudinal range extends from sea-level to 1500 m a.s.1. in cooler, more southerly locations such as Tasmania (Key 1992). *P. vittatum* has an annual life cycle, overwintering in the egg stage. Hatchlings pass through five nymphal stages before emerging as adults. The earliest hatchlings may be observed in the field in late spring, with adults surviving into late autumn in warmer years (Baker 2005). Adults can be macropterous, with functional wings, or brachypterous, incapable of flight (referred to here as winged and wingless, respectively). These forms occur together in almost all populations of *P. vittatum* (Key 1992). The wingless form is most abundant in pastures, while the winged is the more abundant form in areas dominated by shrubs, strips along forest margins, and in gardens (Clark 1967).

Females range in size from 12 to 20 mm long, and males from 10 to 13 mm long (Baker 2005). Individuals range in colour from light, through to dark brown and black, and rarely, green. Individuals can be striped, with two white longitudinal stripes on the dorsal surface, unstriped, or patterned, with very dark lateral surfaces on the pronotum and a light dorsal surface. All colours are manifested in the winged and wingless forms, with the exception of the green morph and the very light patterned form, which are not found in winged grasshoppers. The range of colour morphs can be present within the same population and is set for an individual once it reaches the adult stage (Key 1992).

### Thermal properties of colour morphs

Warming-up curves under known radiation levels were used to test for differences in the thermal qualities of the colour morphs of the wingless grasshopper. Twelve runs of four to six randomly chosen specimens were recorded (total *n* = 59). The results of a typical run are shown in Figure 2. Thermocouples were inserted into the thorax of recently killed grasshoppers, and body temperature was recorded every ten seconds after a 375 W infrared heat lamp, 52 cm above the insects, was turned on. The thermocouples were Type T fine wire copper-constantan thermocouples (diameter of individual wire components 0.2 mm) connected to a Campbell Scientific CR10X Datalogger (www.campbellsci.com) via an AM 1632 Multiplexer and using the LoggerNet 3.2 program and a 107TP reference temperature probe. The radiation level was 565 W m^2^, measured using a Campbell Scientific LI200X pyranometer with a spectral range of 400–1100 nm. The substrate on which the grasshoppers rested was sand glued onto a cardboard base. The surface temperature of the substrate was measured using an exposed thermocouple positioned within 2 cm of the grasshopper. After five minutes, a fan was turned on to generate a wind speed of 4 m/s^-1^, striking laterally across the body, and the temperature was logged for an additional 5 minutes. Wind speed was measured using a Kestral 3000 pocket weather meter (www.kestrelmeters.com) positioned in the centre of the arena, 2 cm above the substrate. The reflectivity of the ground surface was measured by inverting the pyranometer and expressing the value as a fraction of the upright measurement. The colour, weight, and femur length of each specimen tested were recorded.

**Figure 1.**
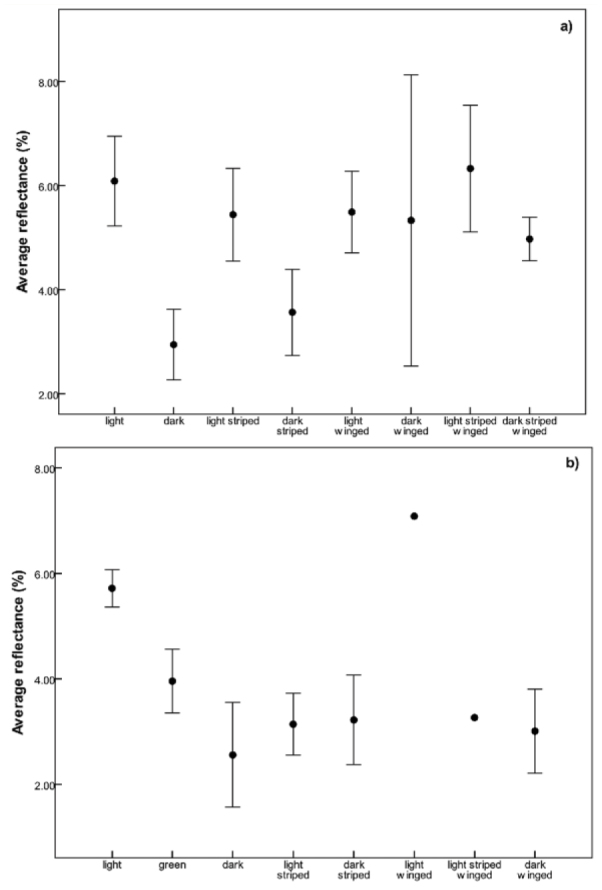
Average reflectance of the colour morphs of a) female and b) male *Phaulacridium vittatum* based on a visual separation of colour morphs. Bars show ± 2 SE. High quality figures are available online.

The relative warming and cooling rates were estimated for each specimen as K = 0.5T_exc_/t_1/2_, where T_exc_ is the temperature excess, the maximum difference between ambient and body temperature that was reached during heating (or cooling), and *t*_1/2_ is the half time of the temperature change (Pereboom and Biesmeijer 2003).

To test whether colour codes could be assigned by eye to colour morphs representing significantly different thermal qualities, each specimen was assigned to a light, medium, or dark category within the striped and unstriped groups. This visual separation into discrete colour morphs was checked against specimens (n = 80) representing the full range of colours in *P. vittatum* (Figure 1). The average reflectance of the light and dark colour morphs wasfound to be significantly different in females (Tukey's q = 3.18 *p* < 0.05) and males (q = 3.31; *p* < 0.05), with the reflectance of the dark categories being significantly higher than the light categories. A strong linear relationship existed between measured reflectance and colour morph (r^2^ = -0.93 for males; r^2^ = 0.75 for females), enabling the results of the warming experiment to be related to the patterns found along the altitudinal gradients.

### Sampling methods

Grasshoppers were collected along 14 altitudinal gradients in Tasmania. On each gradient there were four sites, one in each of the following altitudinal bands:

< 100 m, 200–250 m, 400–450 m, and 700– 800 m a.s.l. Where possible, a fifth site was sampled at 800–1000 m a.s.l. Grasshoppers were collected from roadside verges and clearings beside roads. A hand-held GPS was used to record the latitude, longitude, and altitude of each site.

At each site, grasshoppers were collected by hand and a sweep net for 20 minutes. The number of individuals collected varied across sites, depending on their abundance. At sites where the grasshoppers were very abundant, approximately 20 individuals were collected, and an attempt was made to represent males and females equally. The number of specimens measured at each site was therefore unequal across the sites, ranging from 1 to 25 with a mean number of 12.7 ± 0.56 (Table 1). In total, 853 specimens were measured from the altitudinal gradients within Tasmania, representing 414 females and 439 males. Both winged and wingless grasshoppers were included in the altitudinal analyses because wingless grasshoppers are often scarce at higher altitudes.

### Reflectance

Reflectance is the ratio of reflected to incident light, expressed here as a percentage. The reflectance of each specimen was measured using an Ocean Optics USB2000 spectrophotometer (Ocean Optics Incorporated, www.oceanoptics.com) with a PX-2 pulsed xenon light source. Measurements were taken at an angle of 45°. An attachment was used that reduced the size of the light beam to less than 2 mm in diameter, so that it was completely covered by the body part being measured. The spectrophotometer was connected to a PC running Ocean Optics OOIBase 32 v. 1.0.2.0 software, with the integration time set at 7 ms, and each measurement averaged 10 times by the software (Bruce et al. 2005). All sample reflectance spectra were calculated relative to a Barium sulphate white standard, and a dark and white standard reference spectrum was taken every 10 minutes during measurement of samples. Measurements were taken in a dark room. The range of 300 to 700 nm was used, as this was the range of sensitivity of the machine. Ideally, reflectance would have been measured over the wavelengths 290–2600 nm in order to incorporate the ultraviolet, visible, and infrared parts of the spectrum (Gates 1980; Nussear et al. 2000). However, very few studies have objectively measured reflectance, and our measurements of visible radiation represent a significant improvement on studies in which the continuum of colour has been simplified by arbitrarily separating colours into discrete groups.

All specimens were pinned and dried prior to measurement. Willmer and Unwin (1981) found less than 1% variability when they compared reflectance of freshly killed and dried insects. The reflectance was measured at four different positions on each individual, two on the dorsal surface, one on the side of the thorax, and one on the lateral surface of the right femur. The average proportion of light reflected at 5 nm intervals was calculated for each individual. The mean of these measurements was calculated across all measurements for that individual to obtain an average.

### Body size

Body size was estimated using the length of the right femur, which was measured using handheld vernier callipers (accurate to 0.02 mm). Femur length is closely correlated with body size and other size metrics in grasshoppers (Masaki 1967) and is more reliable than body length, which can change as specimens dry.

### Climate Variables

One of the main predictions of the thermal melanism hypothesis is that reflectance should be positively related to ambient temperature and/or solar radiation. This hypothesis was tested using temporally correspondent climate data for each collecting site. Data were extracted from the Australian Water Availability Project climate dataset (Jones et al. 2007). Annual maximum (T_max_) and minimum (T_min_) temperatures and annual rainfall for the year preceding each collection date were extracted at a spatial resolution of 10 km. In this way, the conditions prevailing from the laying of the egg to adulthood were considered.

### Statistical Methods

**Warming curves.** To take size differences into account while testing for differences in relative warming and cooling rates (K), maximum temperature excess (T_exc_), and half time of the temperature change (t_1/2_), a multiple regression model was fitted with randomisation of modified residuals. The predictor variables included were weight, femur length, and colour code. Tests werecarried out using MiniTab Version 16.1.0 (2010) and the REGRESSRESRAN macro from Butler (2001). All probabilities given refer to those for two-sided randomizations.

**Spectral data.** Principal Components Analysis (PCA) was used to condense the spectral data because it has been shown to be sensitive to slight differences in populations within the same colour category (i.e., that are similar in hue) (Grill and Rush 2000). Data were first standardized to the area under the curve (Grill and Rush 2000) then grouped into 5 nm bins and averaged, resulting in 81 summary values per spectrum. Eigenvalues were calculated from a correlation matrix, and PCAl, PCA2, and PCA3 were used as dependent variables in further statistical analysis. One-way ANOVA was used to detect differences among means of components. Where differences were significant in the ANOVA, TukeyKramer's HSD post hoc comparisons were used to determine which altitude category differed significantly from the others.

**Relationship between reflectance and climate.** Hierarchical partitioning of R^2^ values was used to identify the climate variables with the most independent influence on grasshopper reflectance. The relationship between body size and reflectance was also investigated. This method calculates the proportion of variance explained independently and jointly by each variable (Chevan and Sutherland 1991; MacNally 1996; Walsh and Mac Nally 2005) eliminating spurious conclusions based on joint correlations with other independent variables. Analyses were performed using the R public-domain statistical package (R Project for Statistical Computing release 1.9.0, www.r-project.org).

**Figure 2.**
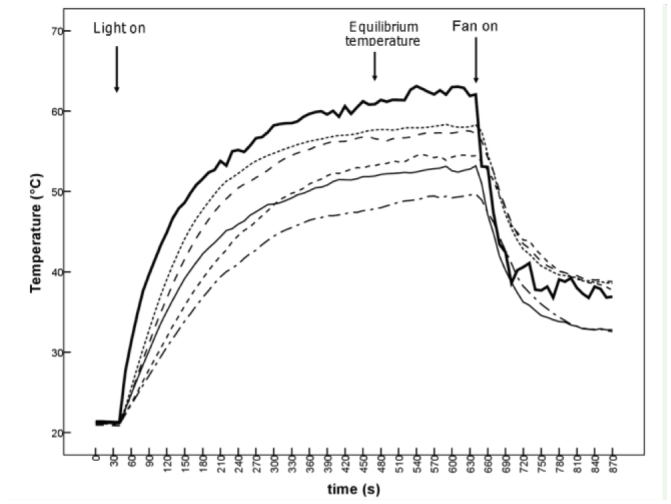
Results of a typical warming run. Ambient temperature is shown by the thick line, and body temperatures of individual *Phaulacridium vittatum* from a range of colour morphs are indicated by the dashed (males) and full (female) lines. High quality figures are available online.

Randomisation was used to quantify relative effect sizes associated with the partitioning by estimating the z-score ([observed - mean {randomisations}] /SD{randomisations}) for each predictor variable, and the statistical significance based on the upper 95% confidence limit (z ≥ 1.65) (MacNally 2002).

## Results

### Warming curves

The typical warming and cooling curves showed that grasshoppers heated up rapidly when exposed to radiation until they reached an equilibrium temperature, and cooled-down rapidly when wind was applied (Figure 2).

The trends predicted by biophysical theory, that smaller and darker grasshoppers warm up more quickly and darker ones reach higher equilibrium temperatures, were generally demonstrated, but the differences were not always significant (Table 2). In unstriped grasshoppers, darker morphs warmed more rapidly than the lightest and reached a higher maximum temperature (lower temperature excess) (Table 3). After weight was accounted for, colour was significantly negatively related to the maximum temperature excess reached (Table 2). Although the relative warming rate was not significantly different between colour morphs, the half time of the temperaturechange (t_1/2_) was significantly different, with darker morphs warming to equilibrium temperature more quickly. Weight was a significant factor in striped grasshoppers, with smaller grasshoppers demonstrating higher temperature excesses. Colour was not a significant factor in this group after weight was accounted for. Cooling rates were not significantly related to colour code or size in striped or unstriped grasshoppers.

### Patterns in reflectance and body size along altitudinal gradients

The average reflectance values for *P. vittatum* ranged between 2.49 and 5.65% (Table 4). Females (n = 414) were significantly lighter than males (n = 439) (F_1,851_ = 56.94, *p* < 0.0001). Striped grasshoppers (n = 203) were slightly lighter than unstriped (n = 650) (F_1,851_ = 8.45, *p* < 0.005). Winged grasshoppers (n = 150) were, on average, significantly darker (F_1,851_ = 14.00, p < 0.0001) and larger (F_1,851_ = 8.15, p < 0.005) than wingless grasshoppers (n = 703).

**Figure 3.**
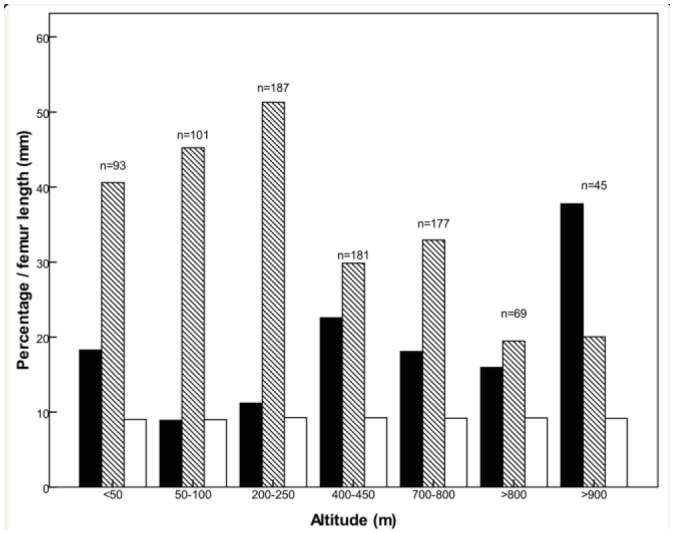
Percentage of winged (black bars) and striped (hatched bars) *Phaulacridium vittatum*, and mean femur length (mm) (open bars) within altitudinal bands. High quality figures are available online.

Overall, average reflectance was negatively correlated with femur length (Spearman's ρ = -0.206, p < 0.01) and negatively correlated with altitude (ρ = -0.104; p < 0.01). So, larger grasshopper were darker, and the higher the altitude, the darker the animals. Over all gradients, the proportion of winged grasshoppers and unstriped morphs increased at higher altitudes (Figure 3). Body size remained constant with altitude (ρ = 0.03, p > 0.05).

Males, when analysed separately from females, did not show a significant relationship between reflectance and size (ρ = 0.040, p > 0.05). However, they did show a marginally significant (ρ = -0.091; p = 0.05) negative correlation between reflectance and altitude. The average reflectance of females showed a significant negative correlation with altitude (ρ = -0.114; p < 0.05), but not femur length (ρ = 0.027, p > 0.05). These correlations were strengthened if sites above 900 m were excluded. Femur length and altitude were not significantly correlated in either males (ρ = 0.031, p > 0.05) or females (ρ = 0.045, p > 0.05).

**Figure 4.**
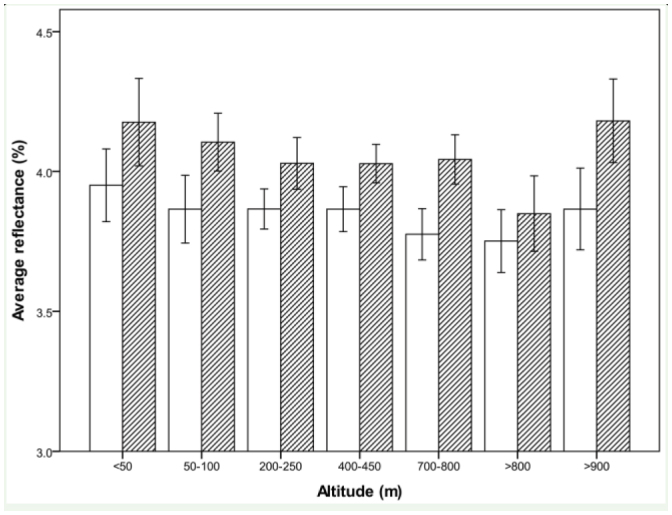
Mean average reflectance of female (open bars) and male (hatched bars) *Phaulacridium vittatum* within altitudinal bands. Bars show ± 2 SE. High quality figures are available online.

On average, grasshoppers collected close to sea level were lighter than those collected between 800 and 900 m a.s.l. (Figure 4). This relationship was significant in male grasshoppers, but not female (Tukey's q = 2.96, p < 0.05). The reflectance then increased at sites above 900 m a.s.l. to close to the same level as at low altitude sites.

**Figure 5.**
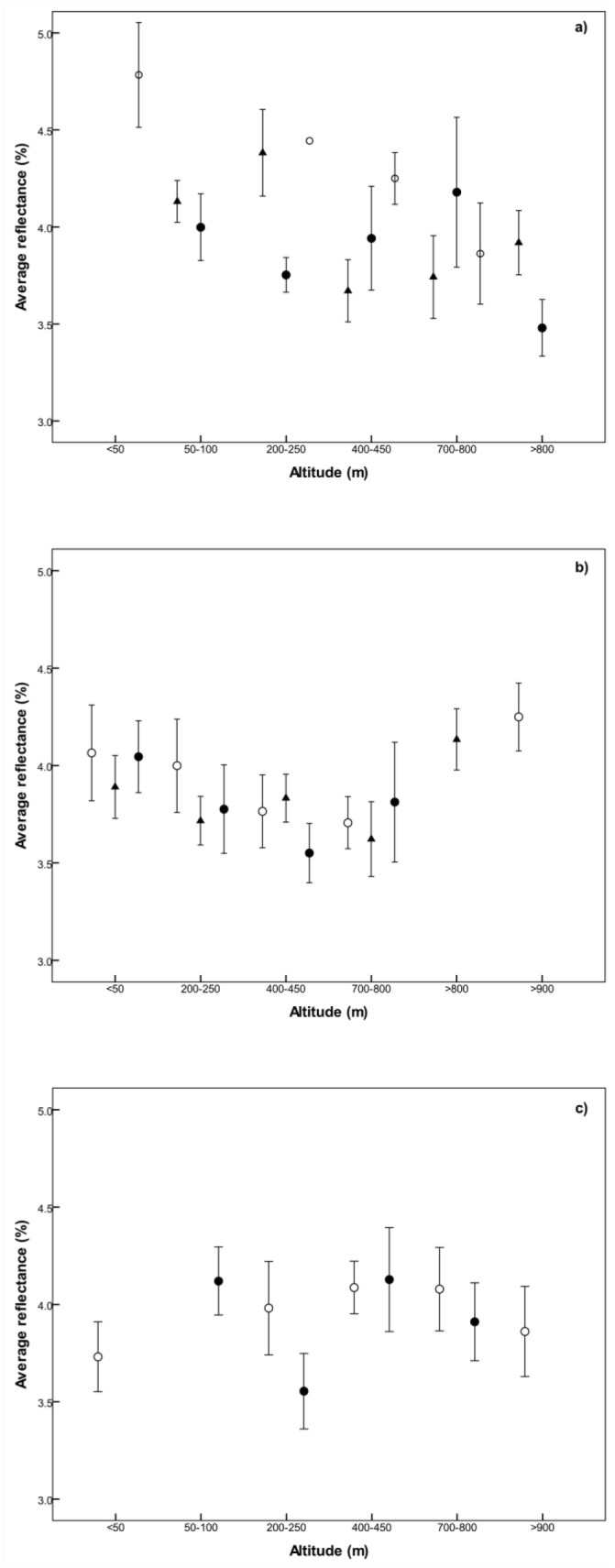
Average reflectance along gradients that showed significant differences between altitudes. a) Gradients on which *Phaulacridium vittatum* were darker at high altitudes compared with low altitudes, b) gradients on which *P. vittatum* became lighter at the highest altitude, c) gradients with site specific patterns. Bars show ± 2 SE. High quality figures are available online.

These trends are based on all the sites combined, but they were not apparent within all gradients. Of the 14 gradients, six did not show any significant difference in average reflectance between sites along the gradient (p > 0.05). On six of the remaining eight gradients, grasshoppers were generally darker at higher altitudes. While the transition was gradual along one gradient, on the others it occurred more sharply above a threshold, which occurred at different altitudes on different gradients (e.g. 400 m a.s.l. on one and 800 m a.s.l. in two other gradients) (Figure 5a). It was only on three of these gradients that grasshoppers became lighter at the highest altitude (Figure 5b). The remaining two gradients showed significant differences that appeared to be site specific (Figure 5c). The differences in reflectance along individual gradients were not related to the number of winged or striped grasshoppers.

### Climatic correlates

The climate variables that explained a significant amount of the variability in average female reflectance were T_max_ (23%) and T_min_ (20%), which were positively correlated with reflectance (Figure 3.6a). Altitude also independently explained 32% of the variability. The negative joint contribution of altitude, femur length, and T_max_ indicates that the majority of the relationships between these variables and the other predictors were suppressive and not additive (Chevan and Sutherland, 1991). The very high value for the joint contribution of T_min_ indicates that this variable was strongly collinear with annual temperature and altitude.

**Figure 6.**
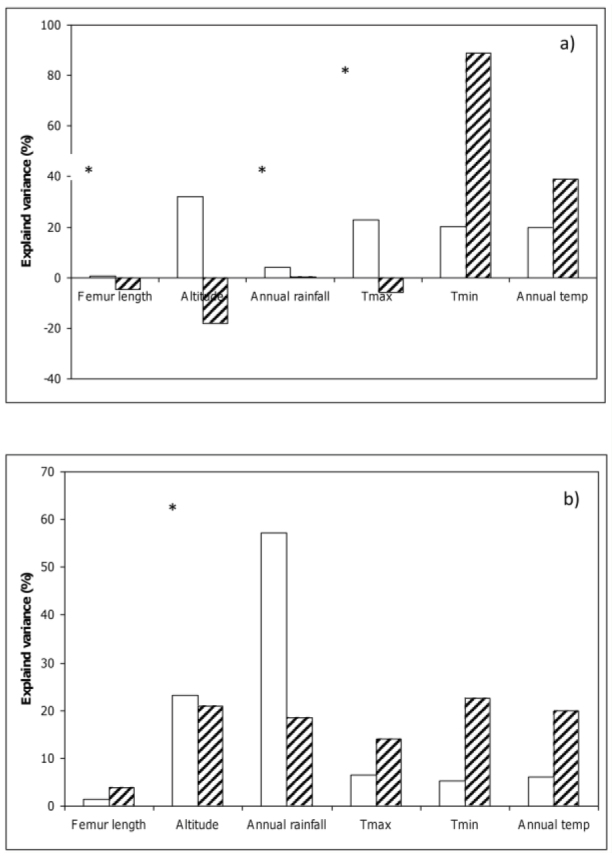
Percentage of variance in the average reflectance of a) female and b) male *Phaulacridium vittatum* explained independently (open bars) and jointly (striped bars) by the climatic variables. Significant independent correlates are indicated by an asterisk. High quality figures are available online.

In contrast to the females, rainfall was the only variable that explained a significant amount of the variability in average male reflectance (57.3%) (Figure 6b). Although altitude explained 21.8% of the variability, it was not a statistically significant contribution.

### Differences in spectra

**Figure 7.**
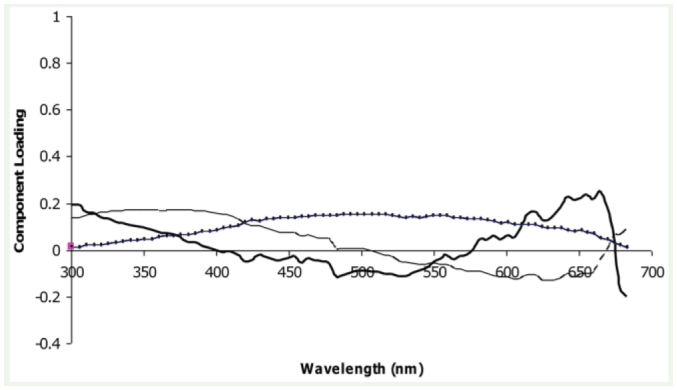
Component loadings from the principle components analysis. PCA1 dotted line, PCA2 fine black line, PCA3 solid black line. High quality figures are available online.

The first three principle components explained approximately 86.95% of the variance in sample reflectance. The principle components can be interpreted by looking at the strength of the loadings across the range of wavelengths analysed and the shape of the spectral curve (Endler 1990; Grill and Rush 2000) (Figure 7). Following Grill and Rush (2000), principle component 1 (PCA1), which explained 47.03% of the variance, was interpreted as being related to the brightness, or overall light intensity of the sample, because the component loadings were relatively even across the range of wavelengths measured. PCA2 explained 32.86% of the variance, and was interpreted as chroma, or the degree to which colour is comprised of neutral white and grey light, because the loadings were strongest at the low and high end of the spectra. Chroma is a function of how rapidly intensity changes with wavelength (Endler 1990). Spectra with steeper maximum slopes and greater differences among parts of the spectra will appear to have more chroma than those with more gradual, smaller changes. The component loadings for PCA3 related to reflectance between 500 and 650 nm, where the slope was greatest; this was interpreted as hue. The hue of a spectrum is a function of its shape, and is directly related to the regions of the spectrum where slope is highest, which usually occur in the middle of a spectrum (Endler 1990; Grill and Rush 2000). This component only explained 7.07% of the variation because all grasshoppers were similar in colour.

The means of all principle components were significantly different between the altitudinal bands (PCA1: F_6,846_ = 5.05; p < 0.0001; PCA2: F_6,846_ = 12.56; p < 0.0001; PC A3: F_6,846_ = 2.21; p < 0.05). Pair-wise comparisons of PCAl showed that brightness was significantly higher at the lowest altitude (<50 m a.s.l) compared with the altitudinal bands 700–800 m a.s.l. and 800–900 m a.s.l. (Tukey's q = 2.96, p < 0.05). There was no difference between this lowest altitude and the highest (> 900 m a.s.l.). Chroma at the highest altitude was significantly higher than at all other altitudes (q = 2.96, p < 0.05). There were no significant pair-wise comparisons in hue.

## Discussion

The thermal melanism hypothesis rests on the assumption that dark individuals are at an advantage under colder conditions compared to light individuals, because they warm more quickly and reach higher equilibrium temperatures (Majerus 1998). In *P. vittatum*, melanism represents a gradation in colour rather than distinct colour morphs, with reflectance ranging from 2.49 to 5.65%. This range in reflectance, although small in magnitude, is sufficient to lead to differences in the thermal qualities of light and dark colour morphs and maintain altitudinal clines in melanism, although habitat differences may weaken the relationship.

### Support for the thermal melanism hypothesis

The magnitude of the differences in thermal qualities between the extreme colour morphs of unstriped grasshoppers is sufficient to affect the thermal balance of these morphs. For example, the maximum temperature excess of the lightest unstriped colour morph ranged from 33.14 to 42.42° C, while that of the darkest unstriped morph ranged from 22.58 to 32.42° C. At low altitudes, where higher surface temperatures are common, dark grasshoppers are therefore disadvantaged because they will spend more time thermoregulating than lighter grasshoppers, and less time on activities such as feeding and mating.

Generally, grasshoppers were darker at higher altitudes, and there was a significant negative correlation between reflectance and altitude in males and females. In support of the thermal melanism hypothesis, maximum and minimum temperature were found to be significant independent influences on the average reflectance of female grasshoppers. That altitude is a significant independent correlate suggests that there is another variable that covaries with altitude, influencing reflectance. If this were UV radiation, which increases by 3 to 7% with 300 m a.s.1. of altitude (Pfeifer et al. 2006), it would further support the thermal melanism hypothesis. Analyses of spectral differences along the altitudinal gradients suggest this is the case. Patterns in chroma were found to be significantly different at the highest altitudes (> 900 m a.s.l.) compared to all other altitudes. While chroma in insects has been linked to camouflage and predation avoidance, it has also been shown to improve UV protection (Endler 1993).

In contrast to females, the average reflectance of males was not significantly influenced by temperature variables. This result is consistent with a broader scale study of reflectance and body size along a latitudinal gradient (Harris et al. 2012). Along the altitudinal gradients, rainfall was the only variable with a statistically significant influence on reflectance. Parkash et al. (2009) found that melanism in *Drosophila* in Australia was related to humidity, which supports the melanismdesiccation hypothesis rather than thermal melanism. Alternatively, and possibly more likely, rainfall could be reflecting cloudiness or radiation levels, which play a primary role in maintaining body temperature in basking ectotherms.

In the striped morphs, the absence of a significant relationship between melanism and thermal characteristics indicates that the existence of stripes is driven by some factor other than thermal conditions, such as predator avoidance (Forsman and Appelqvist 1999).

### Habitat differences

By comparing melanism along many gradients, different patterns and thresholds above which reflectance declined were identified. Along several gradients, differences in melanism became apparent above 800 m a.s.1., while in one the decline occurred above 400 m a.s.1. On several gradients, grasshoppers became darker up to approximately 800 m a.s.1., and then lightened again above 900 m a.s.1. to levels similar to the lowest altitude populations. These sites were more open habitats, where convective cooling due to higher wind speeds would rapidly reduce any thermal advantage to darker animals (de Jong et al. 1996).

Along several of the gradients there was no correlation between average reflectance and altitude. Several factors could be acting to reduce the strength of the altitudinal clines in melanism. First, the distance covered in these gradients was substantially less than that typical of altitudinal studies in the northern hemisphere, which often extend from sealevel to 2000 m a.s.1. However, evidence of thermal melanism has been found elsewhere along short gradients, for example in subantarctic beetles along a gradient covering only 50–600 m a.s.1. (Davies et al. 2007).

Second, measurements of reflectance may be an imperfect predictor of the total reflectivity of the different colour morphs. The visible part of the spectrum, between 400 and 750 nm, represents less than half of the incident solar radiation (Gates 1980). The near- and long-wave infrared region can represent a large proportion of the total reflectivity of an animal, and may represent a different proportion in different colour morphs (Nussear et al. 2000). If this were the case, it would be necessary to measure reflectance across the full spectral range to represent the differences between the colour morphs. Nevertheless, since the relationship between reflectance in the infrared and the visible spectra is most likely to be consistent within the species, and the current purpose was to compare between sites at different altitudes, the reflectance measurements are an improvement on visual allocation to colour morphs.

Additionally, site differences had a strong influence on melanism, as has been demonstrated in other insect groups (Halkka et al. 1975; Boucelham and Raatikainen 1984). The assumption when studying altitudinal gradients is that populations are exposed to predictable variation in temperature, but this is rarely the case. Habitat differences would strongly influence the thermal qualities of a site, so that the microclimatic temperatures experienced by a grasshopper would differ widely across sites. Where high altitude sites represent marginal habitats, the strength of the relationship between size, reflectance, and altitude will change. Population density, habitat patchiness, and connectedness will also affect gene flow and the potential for local adaptation (Hanski and Meyke 2005). Furthermore, the quality of the light within the different habitats would differ with changes to vegetation structure, aspect, and so on. Endler (1993) characterised four major light habitats based on forest structure, and showed how the differences in the light spectra could have wide ranging effects on visibility, signalling, and microhabitat choice in a range of animals. Although average reflectance was not significantly different at the highest altitudes, there were differences in chroma, possibly reflecting such differences.

These observations are not based on quantified assessments of the differences between the sites. To do so without the need for extensive structural measurements of the vegetation, the ambient light spectra at each site could be measured. Differences in the proportion of direct to diffuse sunlight could be measured, as well as the quality of available light under different conditions. These measurements would encapsulate many characteristics of the site, including the extent and makeup of leaves, bark, and substrate type, which determine the spectra of the diffuse light sources, as well as the angle of the sun (Endler 1993). This approach would provide information on the quality of the available light under different conditions, and could easily be translated into available temperatures.

Finally, clinal variation in body size and melanism may be less important if behavioural modifications are the principle adaptations for thermoregulation, and these modifications may not be consistent across habitat types. In patchy environments, where body temperature can be maintained easily by shuttling, behaviour is the least costly method of thermoregulation, so changes to melanism or size are less necessary (Angilletta et al. 2006).

In contrast, the thermal advantage of melanism is likely to be greatest at open sites with high radiation levels. At these sites, behavioural thermoregulation will magnify the effect of slight thermal differences between the colour morphs and maintain the altitudinal clines. The cumulative effect of small thermal differences is likely to have an impact on activity times and duration. This will be further reinforced where the colour morphs have different behavioural and physiological adaptations. For example, if dark grasshoppers at high altitudes bask for longer to achieve a higher preferred temperature, the cline in melanism would be maintained even when the thermal qualities are only slightly different. Samietz et al. (2005) found that grasshoppers from high altitude sites spent significantly more time actively increasing their body temperatures through basking in sunnier positions. In support of this, dark unstriped individuals of *P. vittatum* do have a higher preferred temperature and maintain higher live body temperatures than light morphs (R.M. Harris, unpublished data). The contribution of behaviour to the maintenance of altitudinal clines in melanism deserves further study.

## Conclusions

Many tests of the thermal melanism hypothesis have focussed on species with very distinct colour morphs representing a wide range in melanism. We have tested the thermal melanism hypothesis on a species in which melanism represents a gradation in colour, and in which the range in reflectance is small (2.49 to 5.65%). Patterns in melanism and spectral characteristics along altitudinal gradients in *P. vittatum* broadly support the thermal melanism hypothesis, although site-specific factors also contribute to the expression of the colour polymorphism.

**Table 1.**
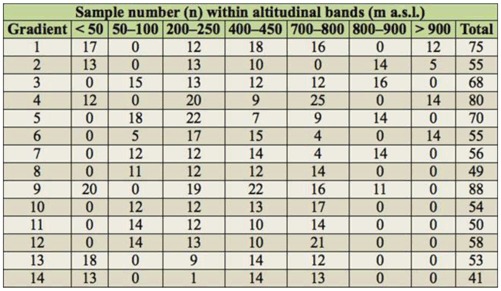
Number of *Phaulacridium vittatum* sampled from each altitudinal band (m a.s.l.) on gradients 1–14.

**Table 2.**
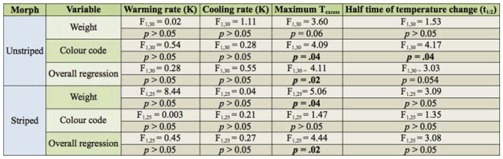
F-ratios and significance of predictor variables in multiple regression. Significant predictors are indicated in bold.

**Table 3.**
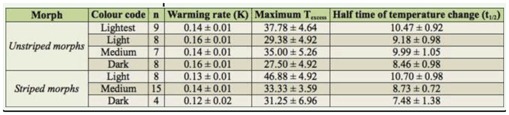
Mean values (± SE) of warming parameters for the different colour morphs of *Phaulacridium vittatum*.

**Table 4.**
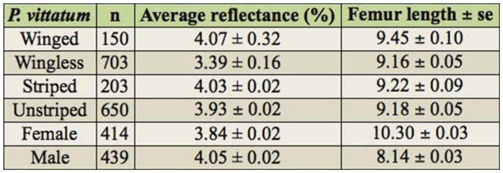
Average reflectance and femur length of *Phaulacridium vittatum*.

## Abbreviations:

PCA,: principal components analysis
